# Role of Adsorbed Polymers on Nanoparticle Dispersion in Drying Polymer Nanocomposite Films

**DOI:** 10.3390/polym13172960

**Published:** 2021-08-31

**Authors:** Sunhyung Kim, Sol Mi Oh, So Youn Kim, Jun Dong Park

**Affiliations:** 1 LG Chem., Corporate R&D, Gwacheon-si 13818, Korea; sunhkim@lgensol.com; 2 School of Energy and Chemical Engineering, Ulsan National Institute of Science and Technology, Ulsan 44919, Korea; ohsolmi82@gmail.com; 3 School of Chemical and Biological Engineering, Institute of Chemical Processes, Seoul National University, Seoul 08826, Korea; soyounkim@snu.ac.kr; 4 Department of Chemical and Biological Engineering, Sookmyung Women’s University, Cheongpa-ro 47-gil 100, Yongsan-gu, Seoul 04310, Korea

**Keywords:** drying, dispersion, polymer nanocomposite, *vertical*-SAXS, polymer adsorption

## Abstract

Polymers adsorbed on nanoparticles (NPs) are important elements that determine the dispersion of NPs in polymer nanocomposite (PNC) films. While previous studies have shown that increasing the number of adsorbed polymers on NPs can improve their dispersion during the drying process, the exact mechanism remained unclear. In this study, we investigated the role of adsorbed polymers in determining the microstructure and dispersion of NPs during the drying process. Investigation of the structural development of NPs using the synchrotron *vertical*-small-angle X-ray scattering technique revealed that increasing polymer adsorption suppresses bonding between the NPs at later stages of drying, when they approach each other and come in contact. On the particle length scale, NPs with large amounts of adsorbed polymers form loose clusters, whereas those with smaller amounts of adsorbed polymers form dense clusters. On the cluster length scale, loose clusters of NPs with large amounts of adsorbed polymers build densely packed aggregates, while dense clusters of NPs with small amounts of adsorbed polymers become organized into loose aggregates. The potential for the quantitative control of NP dispersion in PNC films via modification of polymer adsorption was established in this study.

## 1. Introduction

Polymer nanocomposites (PNCs) are used in a variety of fields, including energy and packaging materials [1,2,3,4,5]. PNCs, which consist of nanoparticles (NPs), polymers, and solvents, exhibit excellent physical properties resulting from the synergistic combination of NPs and polymers. For example, it has been reported that yields and tensile strengths of polymers can be significantly improved by adding NPs to the polymers [6,7]. Additionally, many previous studies have shown that PNC materials exhibit improved optical and electrical performances compared to neat polymers [8]. Since NPs with various features have become ubiquitous, researchers are investigating the effects of the size, shape, and surface characteristics of NPs on the properties of PNCs. An important research goal is to develop design principles for PNCs to achieve the required properties.

One of the most important factors in PNC preparation is the dispersion of the NPs in the polymer matrix. Due to the intrinsic incompatibility between NPs and polymer matrices, obtaining well-dispersed NPs in polymer matrices is quite challenging [9,10]. NPs tend to aggregate owing to large interparticle forces and specific areas [11,12]. To address this issue, polymers are chemically grafted or physically adsorbed onto the surface of NPs. The grafted or adsorbed polymers generate immobilized polymer layers that induce steric hindrance between the NPs. The immobilized layer controls the microstructure and resultant physical properties of the PNC. Many studies have been conducted to develop stable adsorbed layers by changing the chemical properties of the particles or polymers, and to elucidate the governing parameters, such as the adsorption density and sizes of the adsorbed polymers [13,14,15,16].

In many applications, including electrodes in fuel cells [17], photovoltaic solar cells [18], solid electrolytes for Li-ion batteries [4], capacitors [19], membranes [20], and coatings [21], PNCs are processed as thin films by drying. Solution casting is a method frequently used to fabricate PNC films. In this method, the polymers and NPs are dispersed in a solvent, followed by solvent evaporation to concentrate the particles and other components (i.e., the binder and salt in the medium). Thus, particle interactions and the resulting NP structure may be altered during solvent evaporation [22,23,24]. Previous studies have shown evidence of improved NP dispersion by polymer adsorption on the NP surface in solution-casted PNC films [13,25,26,27,28,29,30]. A previous study performed by us revealed that polymer adsorption resulted in improved dispersion of NPs in PNC films, suggesting that adsorbed polymers suppress NP aggregation during drying by introducing steric repulsion between the NPs [31]. However, the mechanism underlying the improvement in NP dispersion in the presence of adsorbed polymers during drying is not clearly understood.

In this study, we investigated the role of adsorbed polymers in improving NP dispersion during drying in solution-casted PNC films. An aqueous dispersion of nano-silica and polyvinyl alcohol (PVA) was employed as a PNC solution model system. Two different PNC solutions with different amounts of adsorbed polymers on the NP surface were prepared by varying the stirring duration [30,32]. The temporal evolution of the NP structures were observed in situ during drying using the synchrotron *vertical*-small-angle X-ray scattering (SAXS) technique [23,33]. Based on the analysis of the changes in the SAXS spectra with respect to the drying time, the structural development along two different length scales related to particle size and cluster size was observed during drying. The polymers adsorbed on the NP surface were found to suppress bonding between the NPs at the later stages of drying. Thus, different amounts of adsorbed polymers on the NP surface led to different film microstructures.

## 2. Materials and Methods

In this study, a mixture of silica and PVA was employed as a model PNC solution for solution casting and drying experiments. PVA (10 wt.%) (Aldrich, molecular weight: (31–50) × 10^3^ mol/g, degree of hydrolysis: 87–88%, and density: 1.27 × 10^3^ kg/m^3^) was dissolved in deionized (DI) water at 353 K for 3 h. An aqueous dispersion of charge-stabilized silica (30 wt.%) (Ludox^®^ HS-30, Aldrich, St. Louis, MO, USA, specific surface area: 220 m^2^/g, and density: 2.37 × 10^3^ kg/m^3^) was used as received. The silica particles were found to have an average diameter, *d*, of 15 nm, with σ/*d* = 0.145 (where σ is the width parameter of the Schulz distribution function) [33] using SAXS measurements. The Silica-PVA PNCs were prepared using the composition: silica:PVA:DI water = 10:4:86 by weight. According to a previous study performed by us, the adsorption of PVA on the silica surface increased gradually with the duration of stirring when the degree of hydrolysis of the PVA used was 87% at pH 10 [31]. The gradual increase in PVA adsorption during stirring at pH 10 was explained by the saponification of the acetate group in the PVA chain under basic conditions [32]. In this study, we prepared silica-PVA PNCs at two different stirring durations of 24 and 120 h to adjust the amounts of PVA adsorbed. The corresponding amounts of PVA adsorbed (Γ) were expected to be 0.03 and 0.09 mg/m^2^, respectively [31]. The PNC solutions corresponding to the stirring durations of 24 and 120 h will henceforth be referred to as Γ0.03 and Γ0.09, respectively. The PNC solutions were stirred at 80 rpm at room temperature (25 °C) using a magnetic stirrer.

**Figure 1:**
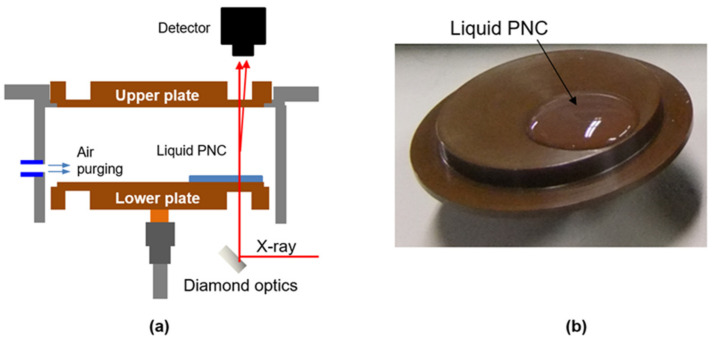
(**a**) Schematics of drying experiment with *vertical*-SAXS. (**b**) Silica-PVA PNC solution loaded onto lower plate.

The drying experiments were performed in conjunction with *vertical*-SAXS measurements on the BW1 beamline at DESY (Hamburg, Germany) [34]. The optical components required for the *vertical*-SAXS measurements were installed in a commercial rheometer (Haake Mars, Thermo scientific, USA) which was specially modified to meet the requirements of the *vertical*-SAXS experiments. The incoming X-ray beam with a wavelength of 1.26 Å is reflected vertically by a specially designed optical system made of diamond and passed successively through a lower plate, the drying suspension, and an upper plate (Figure 1a). The upper and lower plates were made of Vespel (Dupont), which facilitated sufficiently high transmission of X-rays in the applied energy regime of 9.85 keV. The diameter of the lower and upper plates was 38 mm. The PNC solution (0.3 mL) was loaded onto the lower plate using a pipette of diameter 20 mm. The resulting film thickness was approximately 1 mm, assuming cylindrical geometry (Figure 1b). The X-ray beam (area: 400 × 400 μm^2^) was positioned at a distance of 5 mm from the center of the drying sample. The temperature was controlled at 20 ± 2 °C and a relative humidity of 20% ± 5% was maintained by purging the environmental chamber with nitrogen gas at a flow rate of 3 L/min. The in situ SAXS images of the drying colloids were detected using a Pilatus 100 K detector. The sample-to-detector distance was set to 2.43 m, covering a q range of 0.08–0.70 nm^−1^. Time-averaged images were captured for 10 s, at 30 s or 1 min intervals, until images remained invariant, implying complete drying. Further details about the *vertical*-SAXS setup and drying experiments can be found elsewhere [23,33]. We note that the scattering intensity should be measured from the homogeneous microstructure in the thickness direction during the measurement. To find the sample thickness to assure homogeneity during drying, we performed the experiments by varying sample volume over the same area and obtained the reproducible scattering results when sample volume ranged up to 0.3 mL (corresponding to average film thicknesses of 1 mm). Thus, we conducted the drying experiment with a sample amount of 0.3 mL.

## 3. Results and Discussion

### 3.1. Structure of Initial PNC Solution

**Figure 2:**
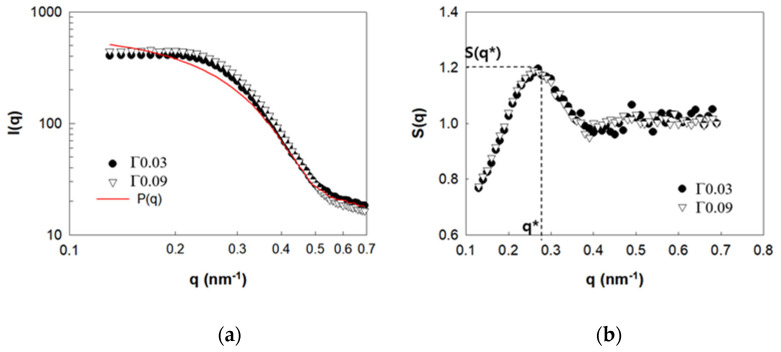
SAXS analysis results of Γ0.03 and Γ0.09. (**a**) I(q) and P(q), (**b**) S(q*).

To understand the effects of the adsorbed polymers on the dispersion states of the NPs in the PNC solutions, the SAXS intensities of Γ0.03 and Γ0.09 were analyzed, as shown in Figure 2. The structure factor, S(q), was obtained by dividing the scattering intensity, I(q), by the form factor, P(q) (S(q)~I(q)/P(q), by invoking the condition that S(q)→1 at high q. Herein, P(q) was determined for the 0.1 wt.% charge-screened silica suspension. The S(q) for both the PNC solutions shows peak height, S(q*) at q* = 0.25 nm^−1^, without upturn at low q, suggesting that the NPs form moderately ordered structures without aggregation. It is plausible that the ordered structures of the NPs resulted from electrostatic repulsion, while the depletion of attraction caused by the non-adsorbed PVA plays a minor role in the dispersion of the NPs. It is of note that S(q) is independent of the amount of adsorbed polymer, indicating that the adsorbed polymer has limited influence on the dispersion of the NPs in the initial PNC solution.

### 3.2. Evolution of SAXS Spectra during Drying

**Figure 3:**
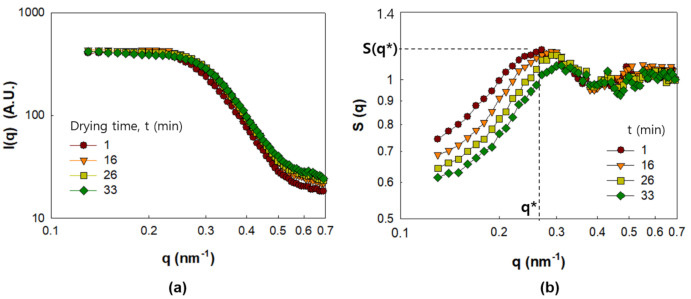
SAXS analysis result of PNC film obtained for Γ0.03 for drying times up to 33 min. (**a**) I(q) and (**b**) S(q*).

In this section, we examine the evolution of NP dispersion in the PNC films corresponding to Γ0.03 and Γ0.09 during drying. The I(q) and S(q) values of Γ0.03 for drying times up to 33 min are displayed in Figure 3a,b, respectively. According to Figure 3b, S(q) shows a peak height S(q*) at q* = 0.25 nm^−1^, which progressively decreases with the shift in the peak position of q* to higher q values, implying that the NPs gradually lose their ordered structures in the PNC films as the solvent evaporates. The gradual development of the NP structures through solvent evaporation can be explained as a result of the increased ionic strength that weakens electrostatic repulsion, and the increasing concentration of non-adsorbed polymers that strengthens depletion attraction [6].

**Figure 4:**
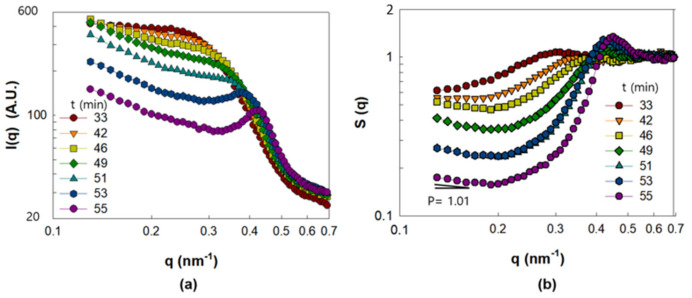
SAXS analysis results of PNC film obtained from Γ0.03 in the range of drying time from 33 to 55 min. (**a**) I(q) and (**b**) S(q).

Figure 4 illustrates the I(q) and S(q) of the PNC film obtained by drying Γ0.03 from 33 to 55 min. I(q) exhibits a change in the slope at low q (Figure 4a) with increasing drying times from t = 33 min to t = 51 min. At low q, I(q) follows power-law behavior, which indicates the formation of a fractal-like aggregated structure of NPs at a large length scale [18]. Furthermore, the shoulder at 0.3 nm^−1^ < q < 0.5 nm^−1^ observed in Figure 4a corresponds to the development of NP structures at a small length scale. The structural evolution of the NPs at the small length scale can be more clearly understood by the analysis of S(q) in Figure 4b. The broad and weak peak height, S(q*), observed at q = 0.28 nm^−1^ and t = 33 min became negligibly small at t = 46 min, which indicates that the structures of the NPs are sparingly ordered. However, the abrupt increase in S(q*) observed from t = 49 min at q = 0.41 nm^−1^ indicates the formation of ordered NP structures at the small length scale corresponding to q* = 0.41 nm^−1^. S(q*) becomes more significant with a slight shift in q* up to q* = 0.45 nm^−1^ at t = 55 min. The inter-particle distance, *d*, calculated from q* = 0.45 nm^−1^ with q*d = 2π, was found to be 14 nm, which is nearly equivalent to the particle size (15 nm). Therefore, the occurrence of S(q*) at t = 49 min and its increase until t = 55 min suggests the formation of dense clusters via contact between some NPs.

**Figure 5:**
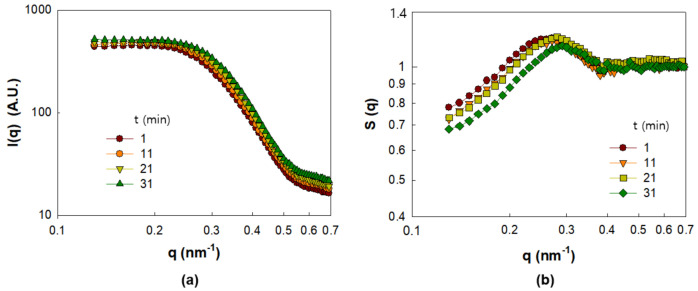
SAXS analysis results of PNC film corresponding to Γ0.09 for drying times up to 31 min. (**a**) I(q) and (**b**) S(q).

Figure 5 shows the evolution of the I(q) and S(q) of the drying PNC film corresponding to Γ0.09 up to t = 31 min of drying time. According to Figure 5b, the S(q) of Γ0.09 shows a gradual reduction with a shift in the peak position, q* = 0.25 nm^−1^, during drying, which is related to a gradual loss of the ordered structure of the NPs. The change in the S(q) of Γ0.09 over time is nearly identical to that of Γ0.03, as shown in Figure 3. It can be concluded from this result that increased amounts of adsorbed polymers do not have a considerable effect on the structure of NPs in the initial drying stage.

**Figure 6:**
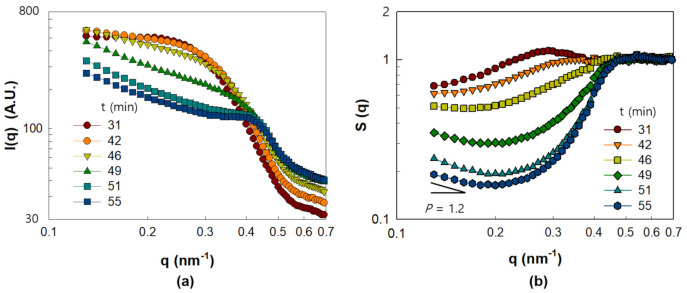
SAXS analysis result of silica-PVA film corresponding to Γ0.09 in the range of drying time from 31 to 55 min. (**a**) I(q) and (**b**) S(q).

Figure 6 illustrates the I(q) and S(q) of the drying PNC film obtained from Γ0.09 in the range of drying time from 31 to 55 min. It is clear from Figure 6b that the S(q) of Γ0.09 does not exhibit any notable peak at high q, which is in contrast with the case of Γ0.03, where an apparent peak height with S(q*) = 1.3 in the q range of 0.4–0.5 nm^−1^ is observed (Figure 4b). Considering that the S(q*) peak at q* = 0.45 nm^−1^ indicates the bonds between the bare surfaces of the NPs, the disappearance of the S(q) peak for Γ0.09 implies decreasing interparticle contact between the NPs due to increased polymer adsorption on the NP surfaces.

According to Figure 6b, the S(q) of Γ0.09 at low q demonstrates remarkable upturns from t = 49 min, as in the case of Γ0.03. As mentioned earlier, the upturns reflect the existence of a fractal structure at large length scales that corresponds to large aggregates of several clusters. To quantify the fractal structure at cluster length scales, the power law slope, *p,* was calculated for the q range from 0.13 to 0.16 nm^−1^, where *p* is expressed by S(q) ~ q^−*p*^. It was observed that at t = 55 min, *p* = 1.2, which was significantly higher than that of Γ0.03 (*p* = 1.01), shown in Figure 4b. Considering that the q range in which the slope, *p,* is calculated corresponds to large aggregates of several cluster sizes, the higher *p* value of Γ0.09 implies that the microstructure on the cluster length scale is denser in Γ0.09 than in Γ0.03. It is noteworthy that while the particle length scale structure is more ordered and denser for Γ0.03, the cluster length scale structure is denser for Γ0.09.

**Figure 7:**
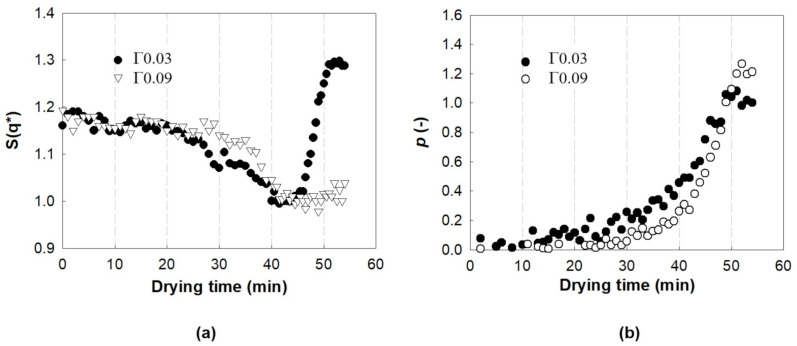
Analysis of I(q) and S(q). (**a**) Peak height, S(q*), of S(q), and (**b**) power law slope of I(q) calculated in the q range between 0.12 and 0.18 nm^−1^.

To extract more information from the SAXS spectra during drying, the I(q) and S(q) were analyzed as functions of drying time, as shown in Figure 7. The S(q*) of both Γ0.03 and Γ0.09 decreases gradually with drying time up to t = 45 min, as shown in Figure 7a. As S(q*) in this initial time range characterizes the ordered structure of the NPs due to electrostatic repulsion, the decrease in S(q*) implies the gradual loss of their ordered structure by solvent evaporation. While the S(q*) of Γ0.03 exhibits an abrupt rise between t = 45 and 52 min, the S(q*) of Γ0.09 does not increase in the same range of drying time. Considering that S(q*) in this time domain corresponds to the contact between the NP surfaces, the absence of such an increase in the S(q*) for Γ0.09 indicates that the increase in the amounts of adsorbed polymers disturbs the bonds between the NPs in the late drying stage, and results in less dense clusters.

For both Γ0.03 and Γ0.09, *p* exhibits an exponential increase with drying time up to t = 51 min, as shown in Figure 7b. Since the *p* value quantifies the fractal dimensions of large aggregates at cluster length scales, the exponential increase in *p* with increasing drying time suggests the emergence of self-similar structures on the cluster length scale. The *p* value of Γ0.09 increases at a slower rate than that of Γ0.03, up to t = 40 min, which signifies that clusters of NPs bearing larger amounts of adsorbed polymers initially form aggregates at a slower rate. However, the *p* value of Γ0.09 rises faster than that of Γ0.03 after t = 40 min, and finally exceeds that of Γ0.03 at the end of drying. The faster and higher increase in the *p* value of Γ0.09 indicates that aggregates on the cluster length scale are formed more rapidly and become denser during the late drying stage of the NPs with larger amounts of adsorbed polymers.

**Figure 8:**
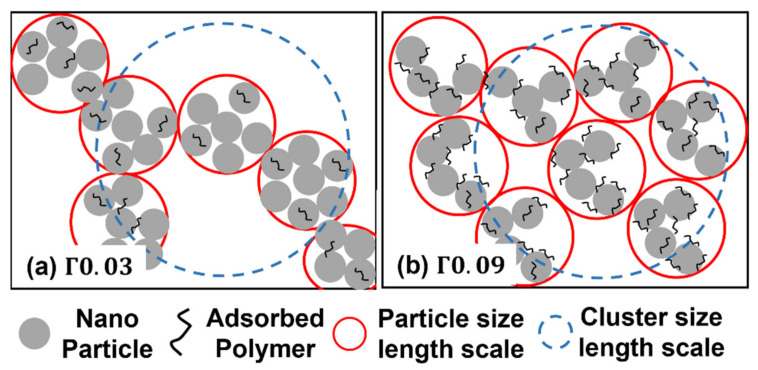
Schematics of SiNP distribution in PVA matrix at t = 55 min. (**a**) Γ0.03 and (**b**) Γ0.09. Red circles: particle length scale corresponding to high q range of SAXS spectra. Blue dashed circles: cluster length scale corresponding to low q range of SAXS spectra.

Compiling all the previous discussions, a schematic explaining the structural development of PNC films and the influence of adsorbed polymers on the NPs during the drying process is illustrated in Figure 8. Based on the S(q) analysis, it has been shown that the polymers adsorbed on the NPs do not play a crucial role in the initial stage of drying. However, as the NPs approach each other and come in contact in the later stage of drying, the polymers adsorbed on the NPs suppress the bonding between the NPs, whereupon the NPs with large amounts of adsorbed polymers form loose clusters with small S(q*), as shown by the red circles in Figure 8b. On the other hand, the NPs with small amounts of adsorbed polymers build dense clusters with large S(q*), as represented by the red circles in Figure 8a. According to the analysis of *p* at low q, different amounts of adsorbed polymers result in different structures on the cluster length scale. The dense clusters consisting of NPs with small amounts of adsorbed polymers (red circle in Figure 8a) are loosely organized into large aggregates (blue dashed circle in Figure 8a), as evidenced by the low value of *p*. On the other hand, loose clusters consisting of NPs with large amounts of adsorbed polymers (red circle in Figure 8b) show relatively large values of *p*, which indicates that the loose clusters are packed to form dense aggregates, as marked by the blue dashed circle in Figure 8b.

**Figure 9:**
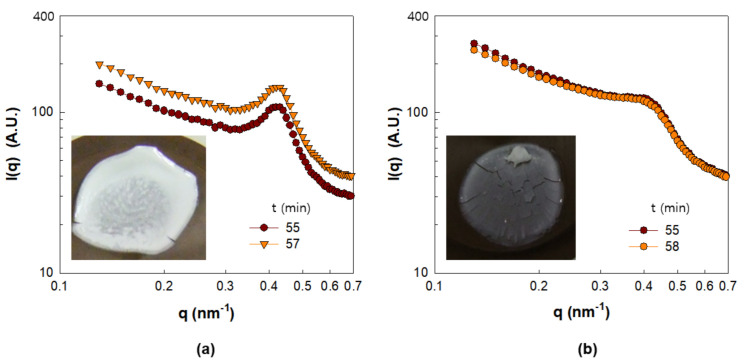
SAXS intensity of silica-PVA film in the range of drying time from 55 to 58 min. (**a**) Γ0.03 and (**b**) Γ0.09.

The study was concluded with an investigation of the dried film structures. Figure 9 shows the I(q) values of Γ0.03 and Γ0.09 beyond t = 55 min. The I(q) spectra do not undergo any change in shape as the structure of the NP dispersion is determined. As can be seen from Figure 9a, the I(q) for Γ0.03 shifted over time. This is attributed to the replacement of water with air between the particles, which causes an increase in the scattering contrast [23,33,35,36]. On the other hand, it can be seen from Figure 9b that the I(q) for Γ0.09 does not shift, suggesting that the intrusion of air does not occur in the PNC film corresponding to Γ0.09. The difference in air intrusion into the PNC films corresponding to Γ0.03 and Γ0.09 is further supported by the enhanced turbidity of the dried film corresponding to Γ0.03 (inset of Figure 9a) compared to that corresponding to Γ0.09 (inset of Figure 9b). Air intrusion into the PNC film enhances the refractive index mismatch between the NPs and the matrix which is responsible for film turbidity [23]. Therefore, the generation of a less turbid film from Γ0.09 suggests that air intrusion between the NPs is suppressed, which can again be explained by the role of increased amounts of adsorbed polymers.

## 4. Conclusions

In this study, the role of polymer adsorption on NPs during PNC film formation through drying has been investigated. In addition, the improvement of NP dispersion in the presence of adsorbed polymers has been examined. Two aqueous dispersions constituting mixtures of nano-silica particles and PVA, which lead to different extents of PVA adsorption, were employed as a model PNC system for performing drying experiments. The structural evolution and dispersion of the NPs in the PNC films were investigated using the *vertical*-SAXS technique.

At the early stage of drying, solvent evaporation leads to an increase in the salt concentration, which gradually renders the NPs in the PNC films unstable due to the loss of electrostatic repulsion. An additional factor that destabilizes the PNC films is the depleted attraction between the NPs caused by the increase in the concentration of non-adsorbed PVA. In the early drying stage, the PNC films bearing different amounts of adsorbed polymers exhibited similar dispersion of NPs. In the late stage of drying when the NPs approach each other and come in contact, the polymers adsorbed on the NPs suppressed the bonding between the NPs. Accordingly, distinctive microstructures manifest in the PNCs depending on the amounts of polymers adsorbed. It was shown that NPs bearing small amounts of adsorbed polymers formed dense clusters on the particle length scale, which further organized into loose aggregates on the cluster length scale. On the contrary, NPs bearing large amounts of adsorbed polymers formed loose clusters that were densely packed on the cluster length scale. Additionally, the adsorbed polymers affected the turbidity of the PNC films by varying the degree of air intrusion between the NPs.

## Data Availability

The data presented in this study are available on request from the corresponding author.
